# Fluorescence Labeling of Peptides: Finding the Optimal Protocol for Coupling Various Dyes to ATCUN-like Structures

**DOI:** 10.1021/acsorginorgau.4c00030

**Published:** 2024-06-04

**Authors:** Jordi C. J. Hintzen, Shitanshu Devrani, Andrew J. Carrod, M. Bahadir Bayik, Daniel Tietze, Alesia A. Tietze

**Affiliations:** † Department of Chemistry and Molecular Biology, Wallenberg Centre for Molecular and Translational Medicine, University of Gothenburg, Medicinaregatan 7B, Gothenburg 413 90, Sweden; ‡ Department of Chemistry and Molecular Biology, University of Gothenburg, Medicinaregatan 7B, Gothenburg 413 90, Sweden

**Keywords:** fluorescence labeling, fluorescence, dyes, peptides, ATCUN

## Abstract

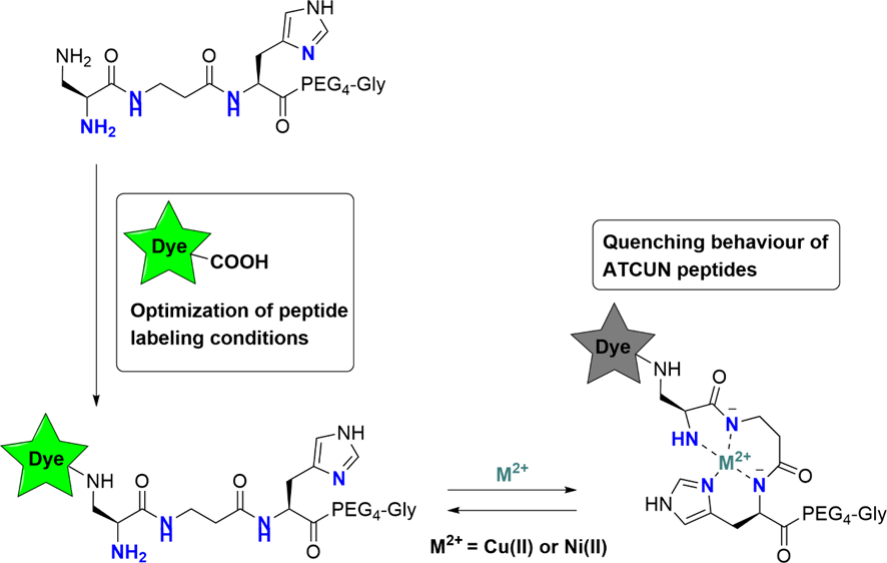

Labeling of peptides and proteins with fluorescent dyes is a key step in functionalizing these structures for a wide array of biological assays. However, coupling strategies of such dyes have not been optimized for the most common compounds, while this step is typically the most precious and costly of the whole synthesis. We searched for the best conditions for attachment of the most widely used fluorescent dyes such as 6-carboxyfluorescein, Rhodamine B, and BODIPY-FL to peptides, where amino terminal Cu(II) and Ni(II) binding site (ATCUN) peptides were used as a model system. Surprisingly, conventional methods of dye attachment proved to not be satisfactory and yielded poor efficiency results. We have discovered that when labeling primary amines on peptides, the uncommon synthesis of activated pentafluorophenol (PFP) esters is the most efficient strategy, expedited by microwave irradiation. Coupling to secondary amines is achieved most efficiently through conventional coupling reagents such as HATU and PyBOP. Furthermore, we have employed our fluorescently labeled ATCUN peptides in studies for Cu(II) and Ni(II) sensing, showing that changing the fluorophore does not significantly affect the fluorescence quenching process and discovering the optimal linker length between the ATCUN core and the dye, expanding the repertoire of fluorophores that can be used in this application.

## Introduction

**Figure 1:**
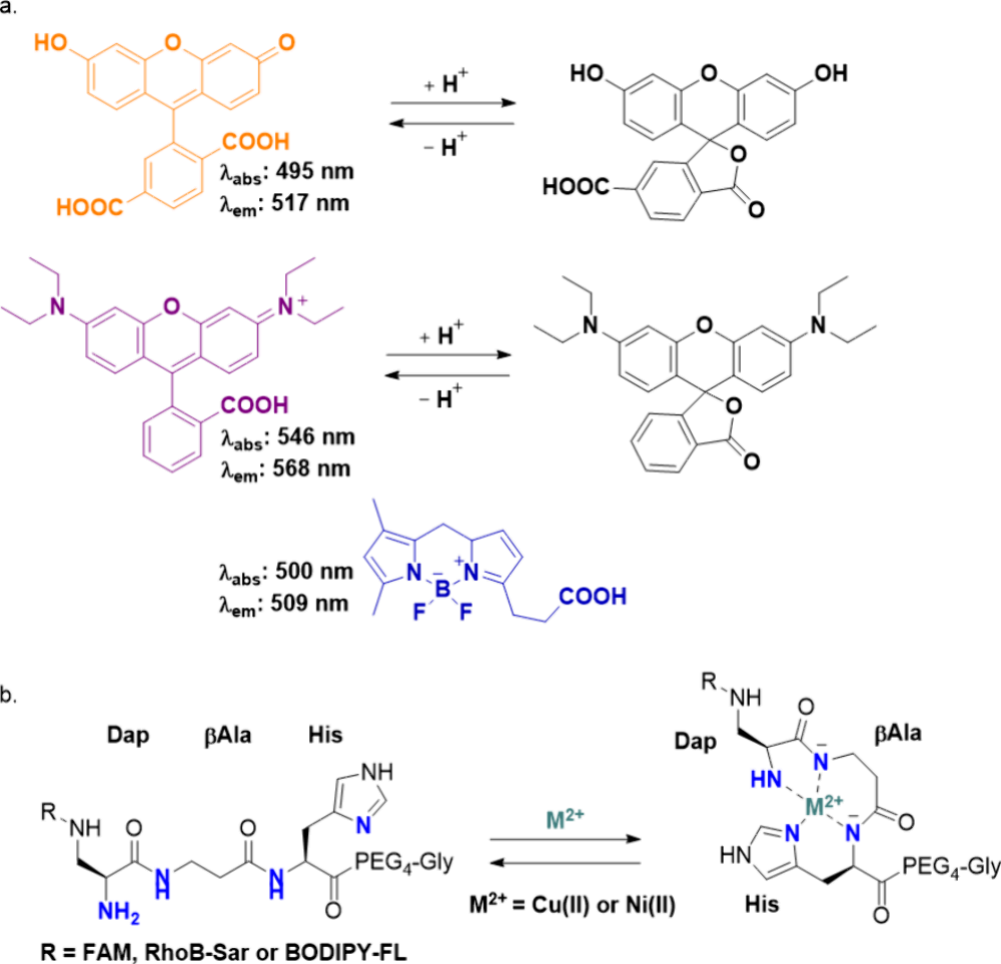
(a) Structures of FAM (top, orange), RhoB (middle, purple), and BODIPY-FL (bottom, blue), including spirolactamization of RhoB and FAM, leading to their respective nonfluorescent isomers, and (b) complexation behavior of the ATCUN motif Dap-β-Ala-His with Cu(II) or Ni(II). The coordinating nitrogens are highlighted in blue.

Fluorescence assays are among the most applied and diverse methods to observe biological processes.^1^ In this regard, the use of fluorescently labeled peptides and proteins provides a facile way to mimic the recognition processes in which these proteins are involved in. Fluorescently labeled proteins can often be obtained by expression-based methods, for example, by fusing a protein of interest to a GFP unit.^2^ However, short peptides can be synthesized and designed with complete freedom to adhere to the specific requirements of an implementation. Synthetic, fluorescent peptides have thus been used in a wide variety of applications, ranging from miniaturized peptide receptors to antimicrobial peptides and cell-penetrating peptides, which can be directly used for cell imaging when linked to a fluorophore.^3^ Some of the most common, cheap, and easy-to-use fluorophores are Rhodamine B (RhoB), 6-carboxyfluorescein (FAM), and BODIPY-FL-propanoic acid, which all possess a carboxylic acid moiety, allowing for convenient incorporation into peptides by amide-bond formation conditions, making them often the choice when peptides require fluorescence labeling (Figure 1a).

RhoB is a cationic xanthene-based dye that is highly water-soluble, finding application as a tracer dye in water as well as a staining dye for certain microorganisms. It is also used frequently in biological imaging studies, as it has its absorption maximum at around 550 nm, while its emission maximum is around 570 nm, making it suitable for biological experiments.^4^ When employing RhoB, especially regarding peptides, it has to be noted that it can exist both in an open fluorescent form and a closed nonfluorescent form by the formation of a spirolactam (Figure 1a).^5,6^ This effect is particularly present under physiological or slightly basic conditions and can result in complications for the analysis of samples. FAM is another xanthene-containing dye, possessing two hydroxyl groups on the xanthene functionality in place of the two diethylamines found on RhoB, slightly changing its fluorescence properties by red-shifting the emission maximum to 517 nm. FAM is a derivative of fluorescein, with an additional carboxylic acid attached to its benzene ring (Figure 1a). When FAM is coupled to the N-terminus of a peptide forming an amide bond, its two carboxylic acids result in the possibility of the formation of two inseparable isomers; however, it was shown that incorporation of FAM can result in an increase in sensitivity compared to the other commonly used fluorescein derivative, fluorescein isothiocyanate (FITC), making it a viable alternative to RhoB and FITC.^7^ Notably, cyclization of FAM has also been reported; however, the pH window under which FAM is in the open, fluorescent form is between 6 and 9, enhancing its applicability in biological assays.^6,8^ 4,4-Difluoro-4-bora-3a,4a-diaza-*s*-indacene (BODIPY) being another widely applied fluorescent core has also found application in many different fields.^9^ Due to their generally relatively sharp fluorescence peaks, high quantum yields, and additional insensitivity to pH and other environmental conditions, prematurely, they have found great application in biological studies and are widely used to label peptides and proteins.^10−12^ For our application, the BODIPY core can be modified to carry a carboxylic acid on one of its pyrrole functionalities, giving rise to BODIPY-FL-propanoic acid (Figure 1a),^13,14^ which can be directly applied for amide bond formation in peptide synthesis.

Thus, far, no definite concise protocol has been established for coupling these dyes to the N-terminus of peptides, with yields often suffering as a result. Therefore, we sought to investigate the effectiveness of some of the most commonly used coupling reagents in peptide synthesis for the coupling of FAM, RhoB, and BODIPY-FL with the aim of establishing a “gold standard” method for peptide labeling with these fluorescent moieties. Subsequently, we have characterized Cu(II) and Ni(II) binding as well as fluorescence properties of the amino terminal Cu(II) and Ni(II) binding site (ATCUN) peptides.

In this study, the ATCUN peptide motif was used as a model system, which is a short peptide motif that is well known for forming highly specific complexes with both Cu(II) and Ni(II) ions (Figure 1b).^15^ Naturally found in proteins such as human serum albumin, the motif coordinates Cu(II) or Ni(II) in a square planar geometry by virtue of its N-terminal primary amine, histidine’s imidazole, and two backbone amines (Figure 1b).^16^ The ATCUN motif can act as a highly selective chemosensor, e.g., for detecting unbound copper in biological samples.^17−20^ Essentially, the ATCUN motif behaves as a turn-off sensor when coupled to a fluorescent molecule, where upon binding of the metal cation the fluorescence decrease is the result of a chelation effect between the excited state of the fluorescent molecule and the metal transition state.^21^ Therefore, the ATCUN peptide often needs to be fluorescently labeled to find application in sensing by quenching its fluorescence.^22^ This, combined with its short sequence, makes it an excellent candidate to evaluate the coupling efficiency of commonly used fluorophores in this study (Figure 1a). Previously, we have applied 5,6-carboxyfluorescein (FAM)-labeled ATCUN peptides, specifically the diaminopropionic acid (Dap)-β-Ala-His motif,^23^ to detect Cu(II) and Ni(II) ions in a nanopore-based approach.^18^

## Results and Discussion

We have evaluated different coupling strategies for fluorescence labeling of ATCUN peptides with commonly used dyes Rhodamine B (RhoB), FAM, and BODIPY. To functionalize the ATCUN peptide with a BODIPY fluorophore, we chose to chemically synthesize BODIPY-FL-propanoic acid according to a previously reported synthetic methodology.^13,14,24^ This particular BODIPY variant carries a carboxylic acid functionality that can be directly applied in peptide coupling and is reported to have enhanced stability in acidic media, making it the ideal candidate for use in solid-phase peptide synthesis.^13^ Initially, we chose three of the most commonly used peptide coupling reagents available, namely, 1-((dimethylamino)(dimethylimino)methyl)-1*H*-[1,2,3]triazolo[4,5-*b*]pyridine 3-oxide hexafluorophosphate (HATU) and 2-(1*H*-benzotriazole-1-yl)-1,1,3,3-tetramethylaminium hexfluorophosphate (HBTU) of the *N*-guanidinium salt family as well as benzotriazol-1-yl-oxytri(pyrrolidino) phosphonium hexafluorophosphate (PyBOP) as a representative of the phosphonium salts.^25^ Among these, HATU is generally considered to give the best coupling yields as well as the least amount of epimerization. Due to unsatisfactory results with modern coupling agents when labeling the ATCUN peptide with FAM, we decided to include pentafluorophenol (PFP), which generates highly activated esters with the carboxylic acid that remains to be coupled, in the panel as well.^26^

**Figure 2:**
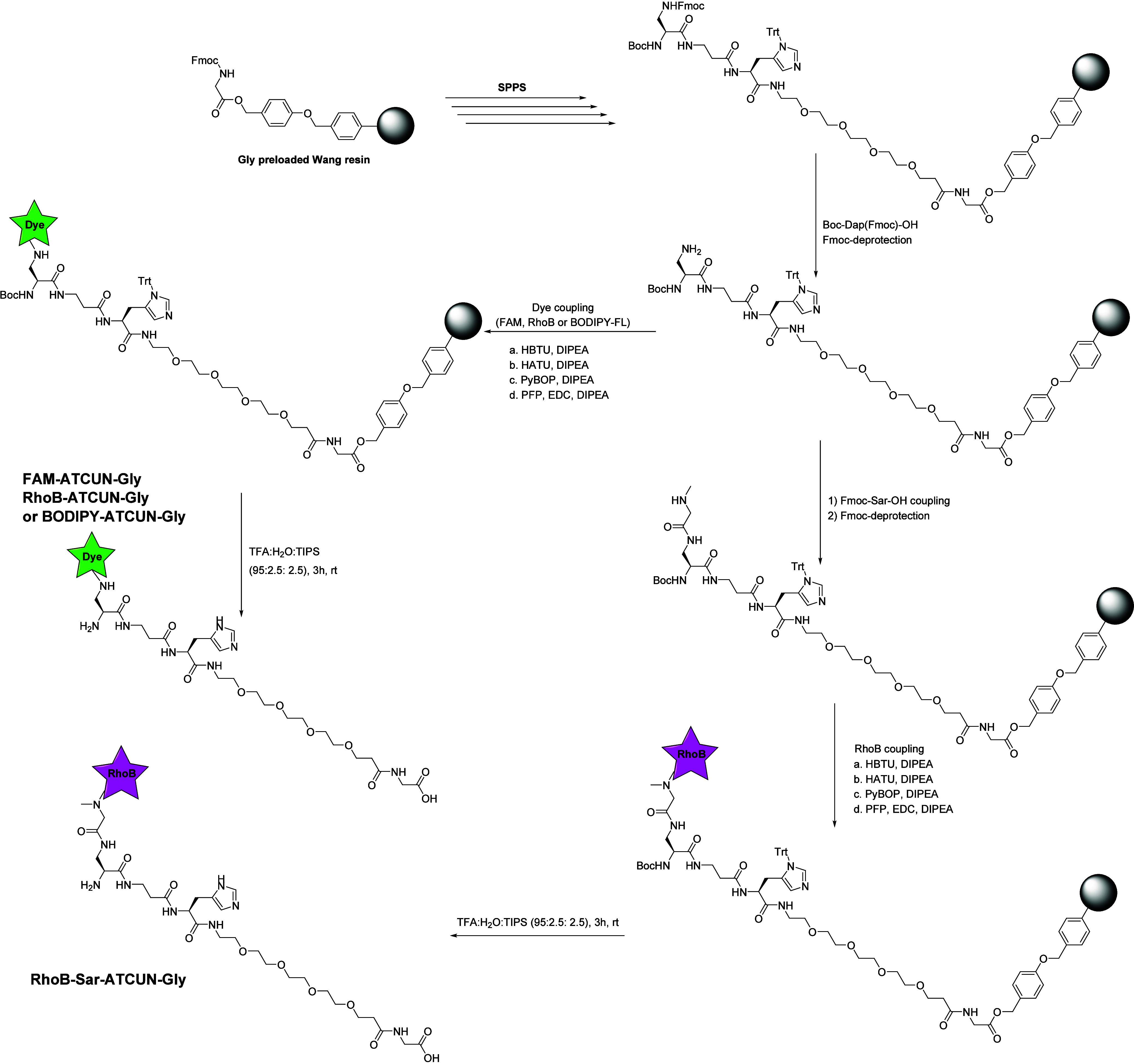
Synthetic scheme of the fluorophore-labeled ATCUN peptides employed in this study. SPPS, solid-phase peptide synthesis; Boc-Dap(Fmoc)-OH, Boc-3-(Fmoc-amino)-l-alanine. Dye represents any of the following fluorophores: FAM, 6-carboxyfluorescein; RhoB, Rhodamine B.

When the FAM-labeled ATCUN peptide was synthesized (Figure 2), conventional coupling agents such as HBTU, HATU, and PyBOP in combination with DIPEA as the base were used. Importantly, to achieve optimal geometry of the metal binding complex,^23^ Boc-Dap(Fmoc)-OH was used so that after deprotection with piperidine, FAM could be coupled to the primary amine of the side chain of Dap, leaving the backbone nitrogen free to coordinate with Cu(II) or Ni(II) (Figure 1b). Surprisingly, we repeatedly observed that for the final FAM labeling step, HBTU and HATU coupling provided very poor yields of the final product, even after double coupling. To potentially alleviate the issues experienced with coupling FAM, four different coupling protocols and reagents (HBTU, HATU, PyBOP, and PFP) were explored, and the efficiency of each of the reagents was systematically evaluated. At this point, note that all preceding coupling steps were performed with HATU and DIPEA as the coupling reagent and base. For HBTU and HATU, the final yield of the purified FAM-ATCUN-Gly was found to be around 3%, whereas for PyBOP, a minor improvement was observed to a 10% yield (Table 1). These yields are not nearly sufficient for a peptide that is to find application in any extensive sensing studies. Thus, we turned to using PFP as a reagent to generate activated carboxylic acids of the FAM fluorophore.^26^ To achieve this, FAM was activated with 1-ethyl-3-(3-(dimethylamino)propyl)carbodiimide (EDC) and, subsequently, the PFP-active ester was generated in the presence of DIPEA and then added to the peptide resin. Fortunately, after cleaving the peptide of the resin and subsequent purification, it was found that PFP was able to efficiently label the ATCUN peptide with FAM, increasing the yield of the overall synthesis to 44% (Table 1), which was a more than satisfactory yield (Figure S1).

**Table 1:** Final Purified Yields for Fluorophore Coupling to ATCUN Peptides Using Different Coupling Reagents

peptide	coupling agent	yield^a^
FAM-ATCUN-Gly	HBTU	3%
	HATU	3%
	PyBOP	10%
	PFP/EDC	**44%**
RhoB-ATCUN-Gly	HBTU	2%
	HATU	25%
	PyBOP	6%
	PFP/EDC	**31%**
BODIPY-ATCUN-Gly	HBTU	Trace
	HATU	1%
	PyBOP	1%
	PFP/EDC	6%
	PFP/EDC (microwave)	**44%**
	TFFH	5%
RhoB-Sar-ATCUN-Gly	HBTU	21%
	HATU	11%
	PyBOP	**29%**
	PFP/EDC	<1%

a Yield calculated over total peptide synthesis (8 steps for FAM-ATCUN-Gly, RhoB-ATCUN-Gly, and BODIPY-ATCUN-Gly or 10 steps for RhoB-Sar-ATCUN-Gly), including purification by preparative HPLC.

**Figure 3:**
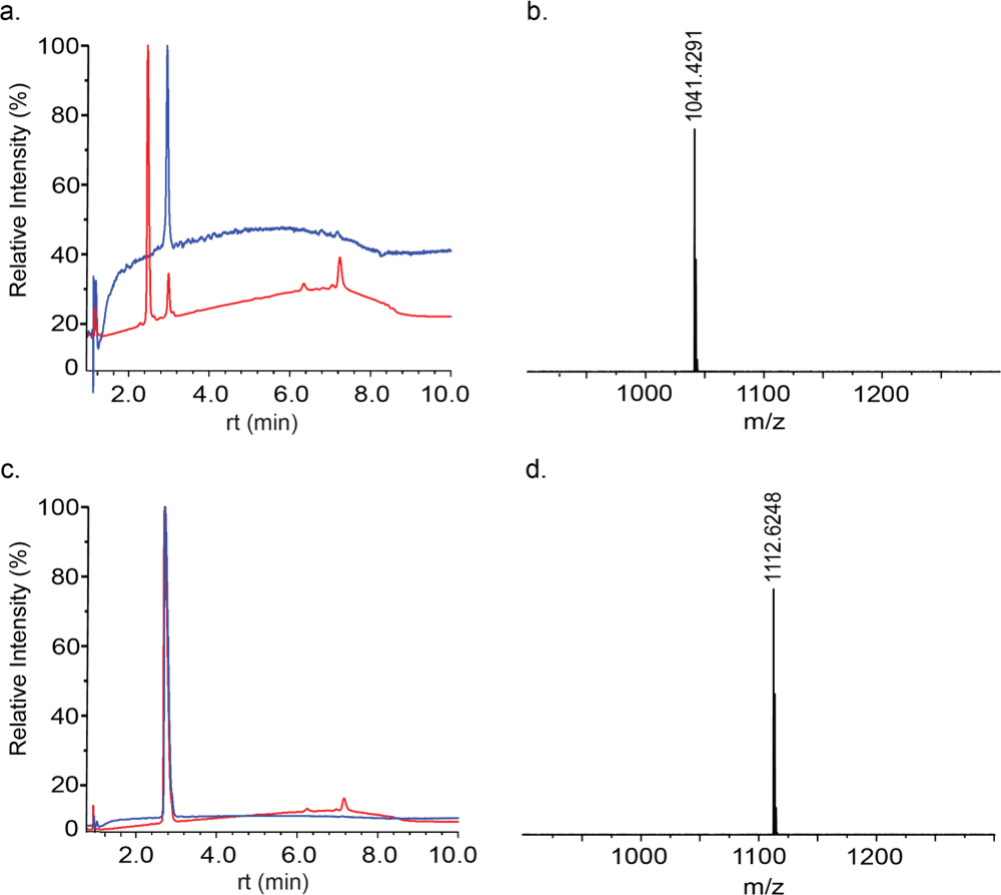
Purification of Rhodamine B-functionalized ATCUN peptides. (a) Analytical HPLC of RhoB-ATCUN-Gly at 214 nm (red) and 540 nm (blue) with a gradient of 5 to 85% ACN over 10 min, (b) LC/MS of RhoB-ATCUN-Gly, (c) analytical HPLC of RhoB-Sar-ATCUN-Gly at 214 nm (red) and 540 nm (blue) with a gradient of 5 to 85% ACN over 10 min, and (d) LC/MS of RhoB-Sar-ATCUN-Gly.

We continued with the synthesis of RhoB-functionalized ATCUN peptides (Figure 2), also with the aim of evaluating RhoB’s behavior in metal binding experiments compared to FAM-labeled ATCUN peptides. Initially, RhoB was coupled to the primary amine of the N-terminal Dap residue; however, any attempt to purify this peptide resulted in the appearance of two distinct peaks in the analytical HPLC spectrum at 214 nm, while the LC/MS data only showed the mass corresponding to the product (Figure 3a,b). When analyzing the same sample through HPLC at 540 nm, which is near the absorption maximum of RhoB, it was observed that only one of the two peaks gave a signal at this wavelength. Therefore, the other peak was suggested to correspond to the RhoB-labeled peptide, which carried the nonfluorescent RhoB isoform (Figure 3a). Indeed, the observed behavior of this peptide in the analytical HPLC could be ascribed to the earlier mentioned spirolactamization of RhoB when adjacent to a primary amine, which would result in a nonfluorescent peptide.^5^ Nonetheless, to be able to comparatively look at the coupling efficiencies of the different coupling agents, we still carried out the synthesis of RhoB-ATCUN-Gly using HATU, HBTU, PyBOP, and PFP, as done for FAM-ATCUN-Gly. Similarly to labeling with FAM, RhoB coupled most efficiently with PFP, albeit with a slightly lower purified yield of 31%, while HATU also resulted in a considerable amount of the final product. In this case, both HBTU and PyBOP gave poor yields, as was found for FAM. The same set of coupling agents was used to link the BODIPY-FL-propanoic acid fluorophore to ATCUN peptides (Figure 2). Surprisingly, overall yields of the peptides were considerably lower than for the coupling studies using FAM and RhoB, indicating that BODIPY-FL-propanoic acid is generally a less efficient fluorophore for peptide labeling purposes. Importantly, the unconventional coupling strategy using PFP yielded, albeit still much poorer than the other fluorophores, the highest efficiency at around 6% overall yield, while the more conventional methods failed to produce more than 1% BODIPY-labeled peptides, obtained for HATU, with trace amounts (<0.1 mg of purified product), HBTU, and PyBOP (Figure S3). Due to the unsatisfactory yields when synthesizing the BODIPY-ATCUN-Gly peptide, we added two additional coupling strategies. First, tetramethylfluoroformamidinium hexafluorophosphate (TFFH) was used as a strategy to generate an alternative carboxylic acid fluoride.^27^ Using TFFH, a final purified yield of 5% was obtained. While it is comparable to the yield obtained with the PFP coupling strategy, it still leaves room for improvement. Second, since PFP still gave the highest yield in the original panel, the BODIPY coupling was reacted under microwave irradiation at 80 °C, which yielded a final purified yield of 44%. These results indicate that PFP is still the most preferable strategy to couple BODIPY-FL, but the dye intrinsically needs slightly harsher conditions for efficient labeling.

To solve the issue of the cyclized RhoB-functionalized peptide, as it appears that the nonfluorescent peak is by far the major product from this initial synthesis (Figure 3a), sarcosine, the smallest *N*-methylated amino acid, was incorporated at the N-terminus and, subsequently, RhoB was coupled to this amino acid (Figure 2). Theoretically, due to the secondary amine of sarcosine’s N-terminus, cyclization of the fluorophore should not occur in this case.^28^ Hypothetically, this introduces the smallest possible change in the ATCUN peptide’s structure while still preventing RhoB cyclization. Fortunately, after purification of the peptide including sarcosine, a single peak was observed in both LC/MS and analytical HPLC at both wavelengths (Figure 3c,d), confirming that RhoB cyclization was efficiently prevented by the inclusion of an *N*-methylated amino acid. Contrary to the results obtained when coupling to the primary amine of Dap, more conventional coupling agents seemed to work best for the coupling of RhoB to the sarcosine N-terminus. Here, similar efficiencies were found with HATU, HBTU, and PyBOP, with PyBOP giving the highest yield over the complete synthesis (29%, Table 1). Additionally, to be able to assess the influence of the distance between the fluorophore and the metal binding center, an ATCUN peptide including two Sar residues between the Dap residue and RhoB was synthesized and purified without any complications (Figure S5).

Using this set of peptide coupling reagents, we have found that the uncommon PFP is the most efficient labeling strategy when coupling FAM, RhoB, and BODIPY-FL-propanoic acid to the primary amine of the peptide’s N-terminus. Interestingly, the PFP coupling method turned out to be nearly completely ineffective when functionalizing RhoB to the secondary amine of sarcosine, which was introduced to prevent RhoB spirolactamization, suggesting that conventional coupling strategies like PyBOP were more efficient. These results highlight the importance of a systematic study of coupling strategies when moving to an alternative fluorophore or peptide terminus, distinguishing clear differences in reactivity between primary and secondary peptide N-termini. Moreover, it is also evident from these results that some dyes can have drastically differentiating coupling efficiencies using the same set of reagents than others, as it becomes evident from the poor yields obtained with BODIPY-FL, raising the importance of careful evaluation of both coupling strategy and choice of fluorophore for a particular purpose, especially when costly, noncommercial fluorophores are being chosen.

**Figure 4:**
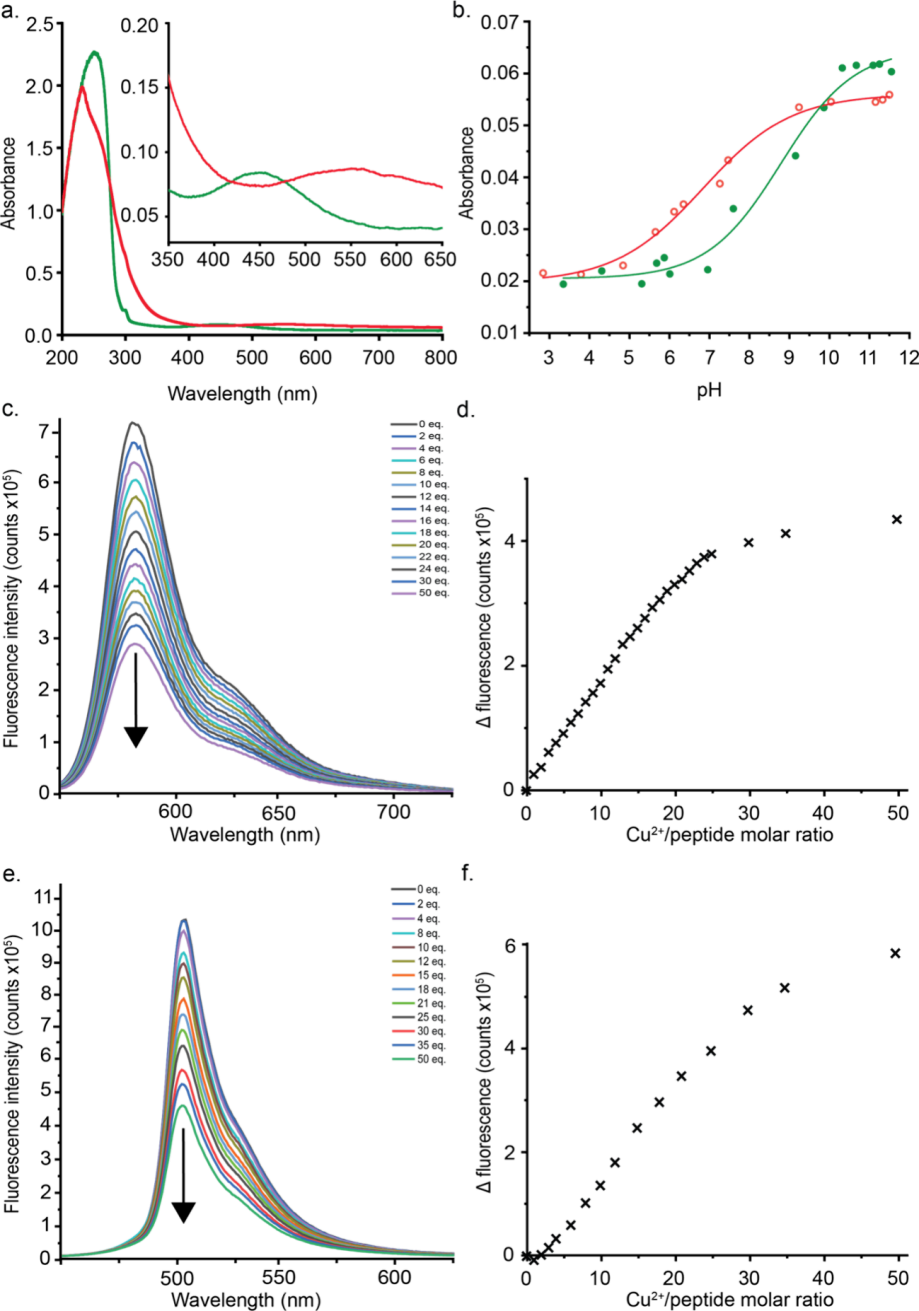
Spectroscopic characterization of metal complex formation of Sar-ATCUN-Gly, RhoB-Sar-ATCUN-Gly, and BODIPY-Sar-ATCUN-Gly. (a) UV/vis spectra of Cu(II) (red) and Ni(II) (green) complexation with Sar-ATCUN-Gly (1 mM, 100 mM Tris buffer, pH 10.5), (b) UV/vis-based spectroscopic determination of the pH dependence of Sar-ATCUN-Gly (1 mM) with Cu(II) (red, 537 nm) and Ni(II) (green, 438 nm), (c) decrease in fluorescence intensity of RhoB-Sar-ATCUN-Gly (5 μM, Tris buffer, pH 9.5) upon addition of CuSO_4_ (0–250 μM, in Tris buffer, pH 9.5), (d) change of fluorescence intensity as a function of the molar ratio of Cu(II) to RhoB-Sar-ATCUN-Gly, (e) decrease in fluorescence intensity of BODIPY-ATCUN-Gly (5 μM, Tris buffer, pH 9.5) upon addition of CuSO_4_ (0–250 μM, in Tris buffer, pH 9.5), and (f) change of fluorescence intensity as a function of the molar ratio of Cu(II) to BODIPY-ATCUN-Gly.

With the RhoB-Sar-ATCUN-Gly and BODIPY-ATCUN-Gly peptides in hand, comparative metal binding assays could be carried out to validate the use of RhoB and BODIPY-FL as fluorophores in fluorescence quenching experiments. The complexation of ATCUN with Cu(II) and Ni(II) is pH-dependent and therefore can be steered by changing the pH of the solution. Previously, it was established that pH 6.5 was ideal for Cu(II) selectivity, while for both metals, the strongest binding was observed at higher pH values.^18^ To confirm whether this important behavior is retained when sarcosine is incorporated in the ATCUN peptide, a UV/vis-based titration experiment was carried out, where the pH of the solution was varied over the range from 3 to 12 in the presence of a 1:1 ratio of ATCUN peptide and Cu(II) or Ni(II). In this assay, a nonfluorescently labeled version of the ATCUN peptide was employed, as the signal of the dye would overlap with the absorbance band observed for the metal ions. Here, an acetylated ATCUN peptide was used in this assay, as the primary amine of the N-terminus can potentially participate in the metal ion coordination.^18^ In this case, sarcosine is the N-terminal amino acid, carrying a methylated N-terminus, thereby alleviating the need for additional N-terminal acetylation due to the secondary amine’s inability to coordinate the metal ion. First, by mixing a 1:1 solution of Sar-ATCUN-Gly and either Ni(II) or Cu(II), clear absorbance peaks at 438 and 537 nm for Ni(II) and Cu(II), respectively, were observed (Figure 4a). These bands are characteristic for the earlier reported d–d transition when square planar complexes between the peptide and the divalent metal ions are formed.^16,18^ Similar to the Ac-ATCUN peptide that was studied earlier, Sar-ATCUN-Gly also displays highly pH-dependent complexation behavior with Ni(II) and Cu(II) (Figure 4b). To our delight, the profiles for both metals followed the same trends for Ac-ATCUN as for Sar-ATCUN-Gly.^18^ This implies that the inclusion of sarcosine at the ATCUN N-terminus does not significantly impact the metal complexation. Importantly, the observed pH dependence still allows for selective detection of Cu(II) ions at a pH near 6.5, as complexation for Cu(II) occurs between 5 and 8.5, while for Ni(II), it is observed between 7 and 10 (Figure 4b).

We subsequently investigated the binding efficiencies of the differently labeled ATCUN peptides with Cu(II) and Ni(II) to assess whether the different fluorescent moieties influence the binding behavior of the metal ions. To be able to probe the binding behavior of both Cu(II) and Ni(II) efficiently during the binding experiments, a pH of 9.5 was chosen to ensure near optimal binding for both ions (Figure 4b). First, UV/vis spectroscopy was used to assay the binding constants of the unlabeled Sar-ATCUN-Gly peptide by analyzing the increase in intensity at 438 and 537 nm for Ni(II) and Cu(II), respectively, over increasing equivalents of metal ions. At pH 9.5, where both ions should efficiently coordinate with the ATCUN core, we found binding constants of 2.65 × 10^6^ and 6.84 × 10^5^ M^–1^ for Cu(II) and Ni(II), respectively (Table 2 and Figures S6 and S7). Comparing these binding constants to the values obtained earlier for the Ac-ATCUN peptide supports the conclusion that the introduction of the C-terminal glycine residue does not significantly affect the binding of metal cations (Table 2).^18^

**Table 2:** Cu(II) and Ni(II) Binding Constants for ATCUN Peptides Were Determined by Fluorescence or UV/Vis Spectroscopy

		pH	*K* (mol/L)	log *K*
fluorescence				
Rhob-Sar-ATCUN-Gly	Cu(II)	9.5	4.32 × 10^12^	12.64
	Ni(II)	9.5	5.75 × 10^12^	12.76
RhoB-Sar-Sar-ATCUN-Gly	Cu(II)	9.5	1.97 × 10^12^	12.29
	Ni(II)	9.5	1.96 × 10^12^	12.29
BODIPY-ATCUN-Gly	Cu(II)	9.5	8.86 × 10^11^	11.94
	Ni(II)	9.5	8.93 × 10^11^	11.95
FAM-ATCUN^18^	Cu(II)	6.5	2.10 × 10^9^	9.32
	Ni(II)	10.5	6.15 × 10^11^	11.79
UV/vis				
Sar-ATCUN-Gly	Cu(II)	9.5	2.65 × 10^6^	6.42
	Ni(II)	9.5	6.84 × 10^5^	5.83
Ac-ATCUN^18^	Cu(II)	6.5	3.8 × 10^6^	6.58
	Ni(II)	10.5	2.5 × 10^7^	7.40

Thereafter, the binding constants of the peptides carrying fluorescent moieties were determined by using fluorescence spectroscopy. It was observed that the fluorescence maximum at 586 nm and the fluorescence lifetime of 1.8 ns for RhoB-Sar-ATCUN-Gly corresponded well to reported literature values for the free dye in ethanol (Figure 4c,d and Figures S8 and S9).^29^ Upon addition of Cu(II) and Ni(II), a clear fluorescence decrease could be observed over the range of added metal ion equivalents, and binding constants could be derived from these curves. The binding constants of 4.32 × 10^12^ and 5.75 × 10^12^ M^–1^ for Cu(II) and Ni(II), respectively, at pH 9.5 corresponded well with previously reported values for binding of FAM-ATCUN, especially since the binding constant for FAM-ATCUN with Cu(II) was determined at the less optimal pH 6.5, while for the Ni(II) binding constant, the values obtained for the RhoB-labeled peptide did not differ more than an order of magnitude (Figure S8).^18^ These results strongly indicate that different dyes can be used in this application, confirming that RhoB is a suitable fluorophore for fluorescence quenching experiments induced by the binding of these metal ions. Along with the synthesis of RhoB-Sar-ATCUN-Gly, a peptide carrying two sarcosine residues in between the ATCUN motif and the RhoB fluorophore was synthesized (Figure S5) to probe the effect of an increased linker length on the quenching behavior in the metal ion complexation. While the emission maximum for RhoB-Sar-Sar-ATCUN-Gly did not shift, it could be observed that fluorescence quenching upon addition of an equivalent of Cu(II) or Ni(II) is significantly less compared to RhoB-Sar-ATCUN-Gly (Figure 4c,d and Figure S10). Comparatively, the relative amount of quenching in the RhoB-Sar-Sar-ATCUN-Gly peptide (56.3% for Cu(II) and 63.8% for Ni(II)) compared to that in the RhoB-Sar-ATCUN-Gly peptide (40.1% for Cu(II) and 40.3% for Ni(II)) was greater. Interestingly, the binding constants for the peptide carrying two sarcosine residues did not get impacted (Table 2 and Figure S10), nor did the fluorescence lifetime of 2.1 ns for this peptide compared to RhoB-Sar-ATCUN-Gly (Figure S9). Combining these results clearly indicates neither the binding of the metal ions to the ATCUN motif nor the properties of the RhoB functionality are affected; however, the efficiency of energy transfer to achieve the fluorescence quenching is greatly affected. Eventually, this results in a generally less sensitive probe and shows the importance of appropriate linker lengths when considering metal ion detection by ATCUN peptides in applications. Similarly, the BODIPY-ATCUN-Gly peptide was complexed with Cu(II) and Ni(II), where it was seen that the emission maximum shifted from 509 to 512 nm, which is only minor compared to literature values for the free acid form of BODIPY-FL (Figure 4e,f).^30^ While the BODIPY-ATCUN-Gly peptide had a longer fluorescence lifetime at 5.6 ns than the RhoB-labeled peptides (Figure S9), the determined binding constants corresponded well among the full panel (Table 2 and Figure S15), with efficient fluorescence quenching observed for the BODIPY-ATCUN-Gly peptide. Importantly, to prevent dilution of the peptides within the sample upon sequential additions of Cu(II) and Ni(II) solutions to affect the observed quenching phenomenon, UV/vis spectra were measured at set points in the assay to confirm that the intensity of the signal did not decrease throughout the assay. Fortunately, in none of the cases, the UV/vis spectra changed, confirming that the observed lower emission is solely due to the metal ion complexation (Figure S11).

## Conclusions

The coupling efficiencies of commonly used peptide synthesis reagents for the N-terminal labeling with carboxylic acid-functionalized fluorophores such as FAM, RhoB, and BODIPY-FL were evaluated. Subsequently, we studied the ability of the fluorescently labeled ATCUN motif to bind Cu(II) and Ni(II) ions. Surprisingly, we found that conventional coupling strategies do not always yield the best results as reagents such as HATU, HBTU, and PyBOP were inefficient in coupling FAM, RhoB, and BODIPY-FL to the ATCUN core when it has a primary amine at its N-terminus. Rather, the generation of the PFP esters was found to be the most optimal strategy for the synthesis of these peptides, possibly with additional microwave irradiation. Caution is required, though, as PyBOP did yield the best results when attaching RhoB to the secondary amine of sarcosine, exemplifying the need for careful evaluation of coupling strategies when synthesizing precious, fluorescently labeled peptides. While preliminary, we observed that coupling of fluorescent dyes to primary amines is best achieved by PFP, while coupling to the secondary amine of sarcosine was most efficient with conventional coupling methods, such as PyBOP. Furthermore, we vouch for the consideration of PFP esters in use in biological labeling approaches as an alternative to commonly used NHS esters, especially when reactions cannot be carried out outside of the window where NHS esters label efficiently, being pH 7.0 to 9.0.^31^ Furthermore, PFP esters are also less susceptible to hydrolysis than NHS esters.^32^ In this article, it was also shown that the previously used FAM fluorophore can be substituted for RhoB and BODIPY-FL to study the complexation of Cu(II) and Ni(II) ions by the ATCUN motif. The pH-dependent distinction in binding was retained when using different fluorophores, giving rise to the opportunity of using a variety of dyes in the selective detection of Cu(II) or Ni(II). Interestingly, clear involvement of the linker length was found, where attaching two sarcosine residues between RhoB and the ATCUN motif resulted in much poorer quenching efficiency, while RhoB with one sarcosine and BODIPY-FL directly linked to the ATCUN motif resulted in efficient quenching. All in all, it can be concluded that diverse fluorescence reporters of metal binding can be attached to functional peptides modularly, but careful consideration of coupling strategy, linker length, and side reactions is needed for generating an efficient synthesis protocol.

## Experimental Section

### Synthesis of BODIPY-FL-propanoic Acid

#### Pyrrole-2-carbaldehyde

*N*,*N*-Dimethylformamide (1.70 mL, 22 mmol, 1.2 equiv) was stirred at 0 °C under argon, and POCl_3_ (2.05 mL, 22 mmol, 1.2 equiv) was slowly added, after which the reaction was allowed to go to room temperature and stirred for an additional 5 min. The solution was cooled down to 0 °C, and 60 mL of anhydrous dichloroethane (DCE) was added. Separately, a solution of pyrrole (1.28 mmol, 18.4 mmol, 1 equiv) was prepared in 60 mL of DCE under argon, subsequently added to the reaction mixture slowly, and refluxed for 15 min in an oil bath. A 100 mL sat. aq. NaOAc solution was then added and refluxed for another 15 min. The reaction was cooled to room temperature, diluted with diethyl ether, and washed with sat. NaHCO_3_. The organic layers were dried with MgSO_4_ and concentrated to afford pyrrole-2-carbaldehyde as a brown oil (14.7 mmol, 1.40 g, 80% yield).

#### Methyl (*E*)-3-(1*H*-Pyrrol-2-yl)acrylate

Pyrrole-2-carbaldehyde (1.40 g, 14.7 mmol) was dissolved in 30 mL of DCM and was combined with a solution of (triphenylphosphoranylidene)acetate (9.78 g, 29.4 mmol, 2 equiv) in 50 mL of DCM under argon. The mixture was then heated to 45 °C using an oil bath, stirred overnight, and evaporated. The residual oil was purified by flash column chromatography (*n*-pentane/EtOAc, 0–50% EtOAc) to afford the methyl (*E*)-3-(1*H*-pyrrol-2-yl)acrylate as a light orange solid (1.03 g, 6.67 mmol, 45% yield). ESI-MS calcd. for C_8_H_9_NO_2_ [M + H]^+^: 152.06; found, 152.19. Spectroscopic data were in accordance with the previously published literature.^13,14^

#### Methyl 3-(1*H*-Pyrrol-2-yl)propanoate

Methyl (*E*)-3-(1*H*-pyrrol-2-yl)acrylate (1.03 g, 6.67 mmol) was dissolved in 30 mL of degassed MeOH under an argon atmosphere, and Pd/C (10% loading, 257 mg, 3.5 mol %) was added to the reaction. Hydrogen gas was then bubbled through the solution under vigorous stirring for 1 h, and the mixture was filtered through Celite and evaporated. Methyl 3-(1*H*-pyrrol-2-yl)propanoate was obtained as a yellow to brown oil (1.02 g, 6.54 mmol, 98% yield) and used without further purification. ESI-MS calcd. for C_8_H_11_NO_2_ [M + H]^+^: 154.08; found, 154.14. Spectroscopic data were in accordance with the previously published literature.^13,14^

#### BODIPY-FL-methyl Propanoate

Methyl 3-(1*H*-pyrrol-2-yl)propanoate (843 mg, 5.50 mmol) and 3,5-dimethylpyrrole-2-carboxaldehyde (782 mg, 5.5 mmol, 1 equiv) were dissolved in 40 mL of DCE under an inert atmosphere. The reaction was cooled to 0 °C, and POCl_3_ (568 μL, 5.55 mmol, 1.1 equiv) was added dropwise and stirred for 3 h at room temperature. DIPEA (4.1 mL, 24.8 mmol, 4.5 equiv) was then added dropwise at 0 °C, and after 20 min of stirring, boron trifluoride-diethyl etherate (BF_3_·OEt_2_, 2.75 mL, 22 mmol, 4 equiv) was added dropwise. The reaction was stirred overnight, and the solvent was removed under reduced pressure. Then, it was redissolved in 200 mL of DCM and filtered over Celite, and the solvent was evaporated. The crude product was purified by column chromatography (*n*-pentane/EtOAc, 20% EtOAc) to yield BODIPY-FL-methyl propanoate as a dark green crystalline solid (998 mg, 3.24 mmol, 59% yield). ESI-MS calcd. for C_15_H_19_BF_2_N_2_O_2_ [M + H]^+^: 309.15; found, 309.21. Spectroscopic data were in accordance with the previously published literature.^13,14^

#### BODIPY-FL-propanoic Acid

BODIPY-FL-methyl propanoate (998 mg, 3.24 mmol) was dissolved in 30 mL of THF, and then 20 mL of water and 10 mL of conc. HCl were added at 0 °C. The reaction was stirred at room temperature for 52 h, until TLC indicated completion of the reaction. The reaction was then extracted with 30 mL of DCM three times, after which the organic extracts were dried with MgSO_4_, filtered, and evaporated to afford BODIPY-FL-propanoic acid as a dark red crystalline solid (923 mg, 3.14 mmol, 94% yield). ^1^H NMR (600 MHz, CDCl_3_) δ 6.89 (d, *J* = 4.0 Hz, 1H), 6.29 (d, *J* = 4.0 Hz, 1H), 6.12 (s, 1H), 3.70 (t, *J* = 6.4 Hz, 1H), 3.59 (t, *J* = 6.6 Hz, 1H), 3.30 (t, *J* = 7.6 Hz, 2H), 2.83 (t, *J* = 7.6 Hz, 2H), 2.57 (s, 3H), 2.25 (s, 3H), 1.97–1.69 (m, 1H). ESI-MS calcd. for C_14_H_17_BF_2_N_2_O_2_ [M + H]^+^: 295.14; found, 295.13.

### Synthesis and Purification of ATCUN Peptides

Peptides were manually chain assembled on a Gly-preloaded Wang resin. Amino acid couplings were carried out with the molar ratio of (4):(4):(8) of (Fmoc-protected amino acid):(HATU):(DIPEA) at room temperature for 60 min, and to ensure complete reactions, all couplings were carried out twice. Deprotection was achieved in 20% (v/v) piperidine in DMF for 30 min. Coupling of the fluorophores (FAM, RhoB, and BODIPY-FL-propanoic acid) was achieved using either (4):(4):(8) of (fluorophore):(HATU/HBTU/PyBOP):(DIPEA) for 60 min twice or by preactivating the fluorophore (4 equiv) with EDC (4 equiv), PFP (4 equiv), and DIPEA (8 equiv) for 30 min and subsequently adding the mixture to the resin and reacting for 60 min. Additionally, BODIPY-FL-propanoic acid was coupled by preactivating PFP (4 equiv) with EDC (4 equiv) for 30 min and subsequently adding the fluorophore (4 equiv) and DIPEA (8 equiv) to the resin and reacting for 60 min at 80 °C under microwave irradiation or by preactivating TFFH (4 equiv) and DIPEA (8 equiv) for 12 min and reacting for 60 min with the resin. Peptides proceeded to standard cleavage from resin using a mixture of TFA (95%), H_2_O (2.5%), and TIPS (2.5%) for 3 h at room temperature. TFA was removed using N_2_, and the resultant residue was suspended in ice-cold diethyl ether. The mixture was then centrifuged (5 min, 4600 rpm) after which the supernatant was decanted into the waste. The remaining solid was washed twice with ice-cold diethyl ether and subjected to purification. For the purification and characterization of the peptides, two eluent systems were used. Mobile phase A was 0.1% TFA in MQ-H_2_O, mobile phase B was 0.1% TFA in ACN, and detection was done at 214 nm. The crude peptide was dissolved in a mixture of ACN in H_2_O and purified by semipreparative HPLC using a Waters 600 system (Waters, Milford, MA, USA) equipped with a C18 column (MultoKrom 100-5 C18, 5 μm particle size, 100 Å pore size, 250 × 20 mm, CS Chromatographie Service, Langerwehe, Germany) and a gradient of mobile phase A and mobile phase B from 5 to 15% B to 35–45% B over 45 min at 8 mL/min. Analytical RP-HPLC was carried out on a Waters XC e2695 system (Waters, Milford, MA, USA) employing a Waters PDA 2998 diode array detector equipped with an ISAspher 100-3 C18 (C18, 3.0 μm particle size, 100 Å pore size, 50 × 4.6 mm, Isera GmbH, Düren, Germany) at a flow rate of 2 mL/min using a gradient of mobile phase A and mobile phase B from 5 to 85% B over 10 min at 2 mL/min. The molecular weight of the purified peptides was confirmed by ESI mass on a Waters Synapt G2-Si ESI mass spectrometer equipped with a Waters Acquity UPLC system using a Xela C18 column (C18, 1.7 μm particle size, 80 Å pore size, 50 × 3.0 mm, Isera GmbH, Düren, Germany).

### Cu(II)/Ni(II) Complexation Experiments

In all experiments, stocks of Cu(II) and Ni(II) were prepared by using CuSO_4_ and NiCl_2_, respectively. pH titration studies were performed using a 1 mM Sar-ATCUN-Gly solution in deionized water at 25 °C. One equivalent of the according metal ion, Cu(II) or Ni(II), was added, and the pH lowered to pH < 3 using 0.1 mM HCl. By adding aliquots of 0.1 mM NaOH, the pH was increased and, with each step, a spectrum was recorded. The absorbance values at 438 nm for Cu(II) and 537 nm for Ni(II) were then plotted against the pH. UV/vis titration to determine the binding constants of Sar-ATCUN-Gly was performed using a stock of 1 mM Sar-ATCUN-Gly in Tris buffer (pH 9.5), and aliquots of Cu(II) and Ni(II) in Tris buffer (pH 9.5) were added over the range of 0 to 1.6 mM and plotted against the absorbance. For fluorescence-based binding constant determination, stocks of RhoB-Sar-ATCUN-Gly, RhoB-Sar-Sar-ATCUN-Gly, and BODIPY-ATCUN-Gly were prepared at 5 μM in Tris buffer (pH 9.5). Aliquots of Cu(II) and Ni(II) in Tris buffer were added over the range of 0 to 250 μM. To ensure avoiding dilution of the peptides, an equivalent amount of peptide was added with each addition, and UV/vis spectra were measured at 0, 12, 25, and 50 equiv of Cu(II) or Ni(II). To determine the binding constants, the decrease in fluorescence at 586 and 512 nm for RhoB-labeled or BODIPY-labeled peptides, respectively, were plotted against the equivalents of Cu(II) and Ni(II) added. For all binding constants, Stern–Volmer fitting was applied over the linear area of the curve.^33^ Fluorescence decay was recorded by time-correlated single photon counting (TCSPC) on the same spectrofluorometer, with a picosecond pulsed diode laser (475 or 510 nm) as the excitation source and an MCP-PMT as the detector.

## Data Availability

The data underlying this study are available in the published article and its Supporting Information.

## Supporting Information Available

Materials and methods, analytical data for peptides, NMR data, and supporting figures of UV/vis titration experiments, fluorescence spectra, fluorescence lifetime measurements, and Stern–Volmer binding curve plots (PDF)
