# Supplementary material for: Fluorescence Labeling of Peptides: Finding the Optimal Protocol for Coupling Various Dyes to ATCUN-like Structures
Source: ACS Org Inorg Au. 2024 Jun 4;4(5):517–25. doi: 10.1021/acsorginorgau.4c00030 (PMC11450724; doi:10.1021/acsorginorgau.4c00030)
Supplement: Supplementary file 1 — gg4c00030_si_001.pdf [file gg4c00030_si_001.pdf]

Supporting information

Fluorescent labeling of peptides: finding the optimal protocol for coupling various dyes to ATCUN-like Structures

Jordi C. J. Hintzen,[a#] Shitanshu Devrani,[a#] Andrew J. Carrod,[b] M. Bahadir Bayik,[a] Daniel Tietze,[a] and Alesia A. Tietze[a,*]

[a] University of Gothenburg, Department of Chemistry and Molecular Biology, Wallenberg Centre for Molecular and Translational Medicine, Medicinaregatan 7B, 413 90 Gothenburg, Sweden

[b] University of Gothenburg, Department of Chemistry and Molecular Biology, Medicinaregatan 7B, 413 90 Gothenburg, Sweden

# these authors contributed equally to this work

Materials and methods

All amino acid derivatives were purchased from Iris Biotech GmbH and used without further purification. ***Novabiochem***® Wang resin served as the solid support for the synthesis of carboxyl group at the C-terminal. All the resins were obtained from Merck. All solvents and reagents were purchased from commercial sources and used as received. Photophysical measurements were carried out in polished 1 cm path length quartz cuvettes. UV-Visible absorption spectra of dye modified peptides were recorded on a Perkin Elmer LAMBDA 950 spectrometer. Steady state emission spectra were measured on an Edinburgh Instruments FLS 1000 spectrofluorometer with a 450W Xenon arc lamp as the excitation source, and a double monochromator to select the wavelengths of excitation and emission. Fluorescence decay was recorded by time-correlated single photon counting (TCSPC) on the same spectrofluorometer, with a picosecond pulsed diode laser (475 or 510 nm) as the excitation source and an MCP-PMT as detector.

Table S1. Analytical data for the peptides

Number	Peptide	Calculated Mw [g/mol]	Experimental[a] Mw [g/mol]	ppm	tR [min]
**1**	FAM-ATCUN-Gly	975.3736	975.3693	-4.4	1.62
**2**	Sar-ATCUN-Gly	688.3630	688.3632	0.3	3.39
**3**	RhoB-ATCUN-Gly	1042.5488	1042.5420	-6.5	2.14 & 2.89[b]
**4**	RhoB-Sar-ATCUN-Gly	1113.5859	1113.5766	-8.3	2.74
**5**	RhoB-Sar-Sar-ATCUN-Gly	1184.6390	1184.6359	-2.6	4.96
**6**	BODIPY-ATCUN-Gly	891.4433	891.4388	5.0	1.24

[a] Mass peaks detected as $[M+H]^+$
[b] Peak reported for peptide containing non-cyclized and cyclized RhoB

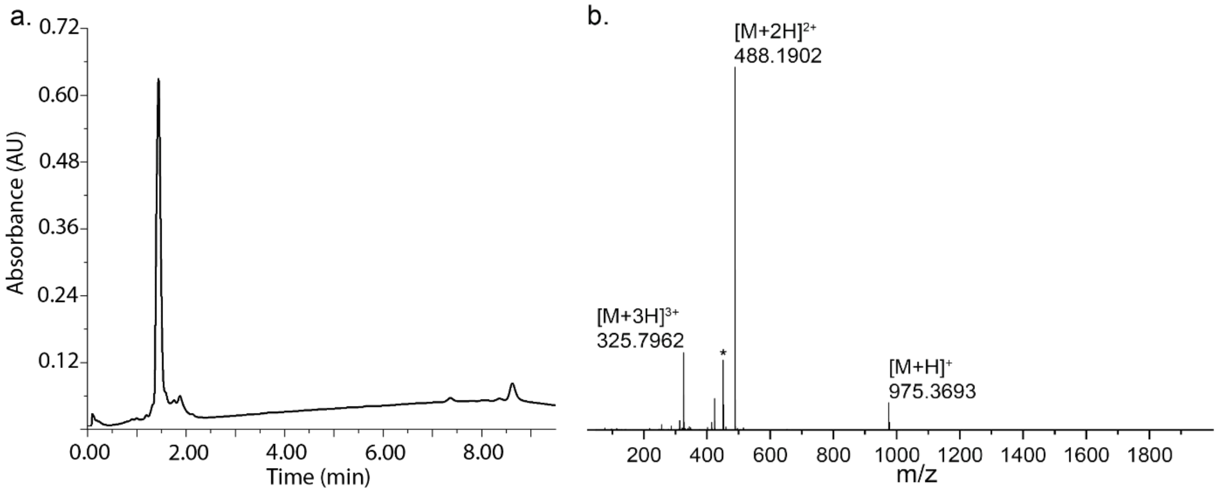

Figure **S1**. **a)** HPLC chromatogram (gradient from 5 to 85% ACN in water over 10 min at 2 ml/min, detection at 214 nm) and **b)** high resolution mass spectrum for FAM-ATCUN-Gly. * impurity from the LC-MS system.

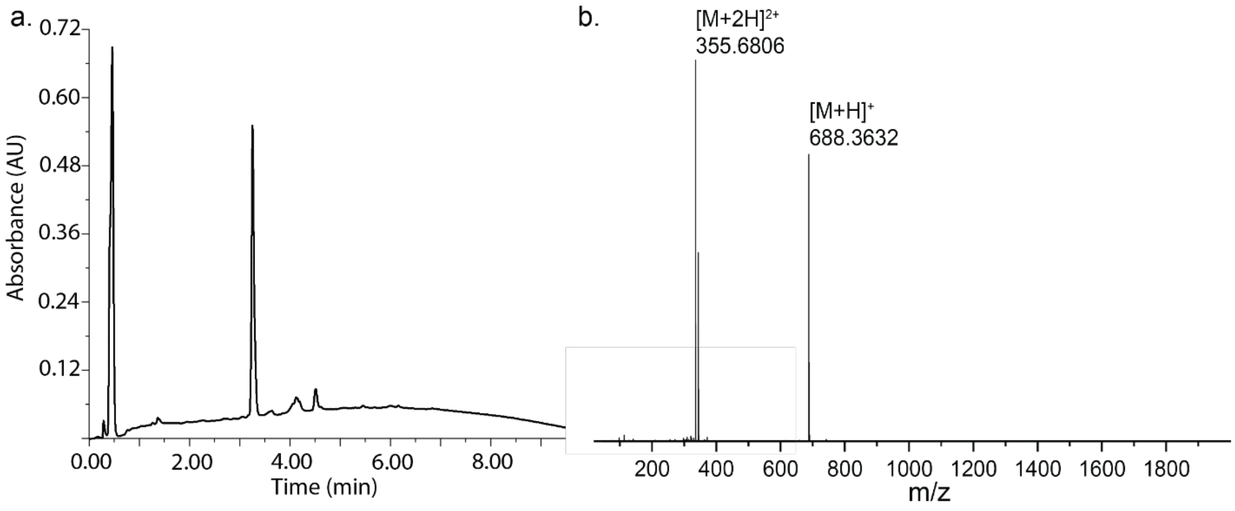

Figure **S2**. **a)** HPLC chromatogram (gradient from 0 to 25% ACN in water over 10 min at 2 ml/min, detection at 214 nm) and **b)** high resolution mass spectrum for Sar-ATCUN-Gly.

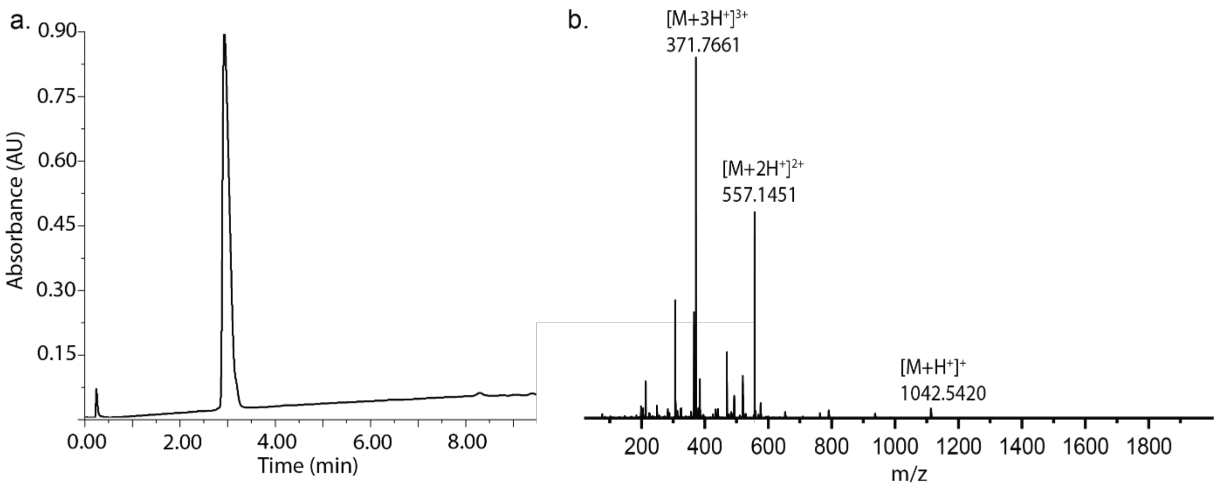

Figure **S3**. **a)** HPLC chromatogram (gradient from 5 to 85% ACN in water over 10 min at 2 ml/min, detection at 214 nm) and **b)** high resolution mass spectrum for RhoB-Sar-ATCUN-Gly.

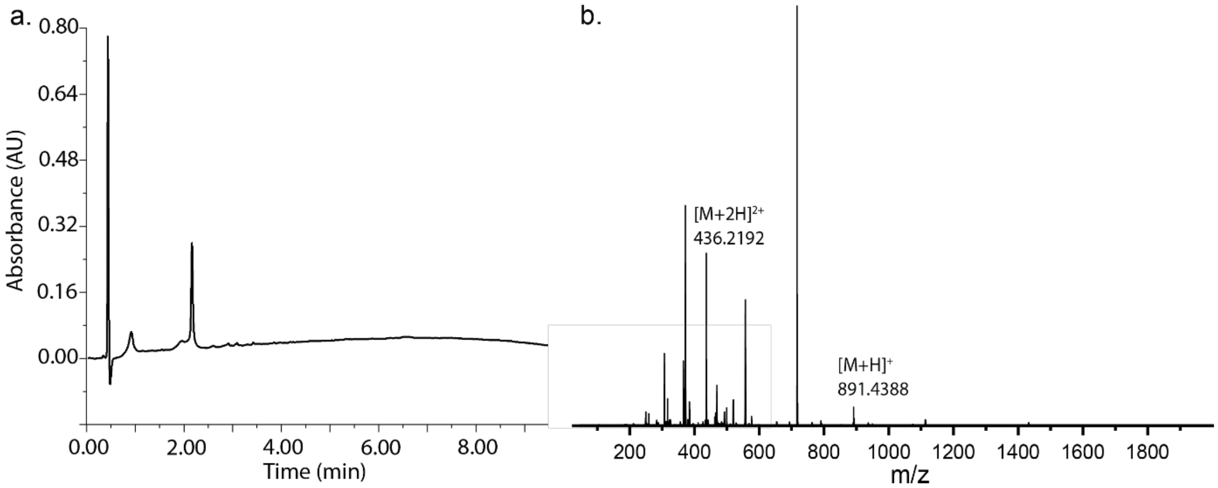

Figure **S4**. **a)** HPLC chromatogram (gradient from 0 to 60% ACN in water over 10 min at 2 ml/min, detection at 214 nm) and **b)** high resolution mass spectrum for BODIPY-ATCUN-Gly.

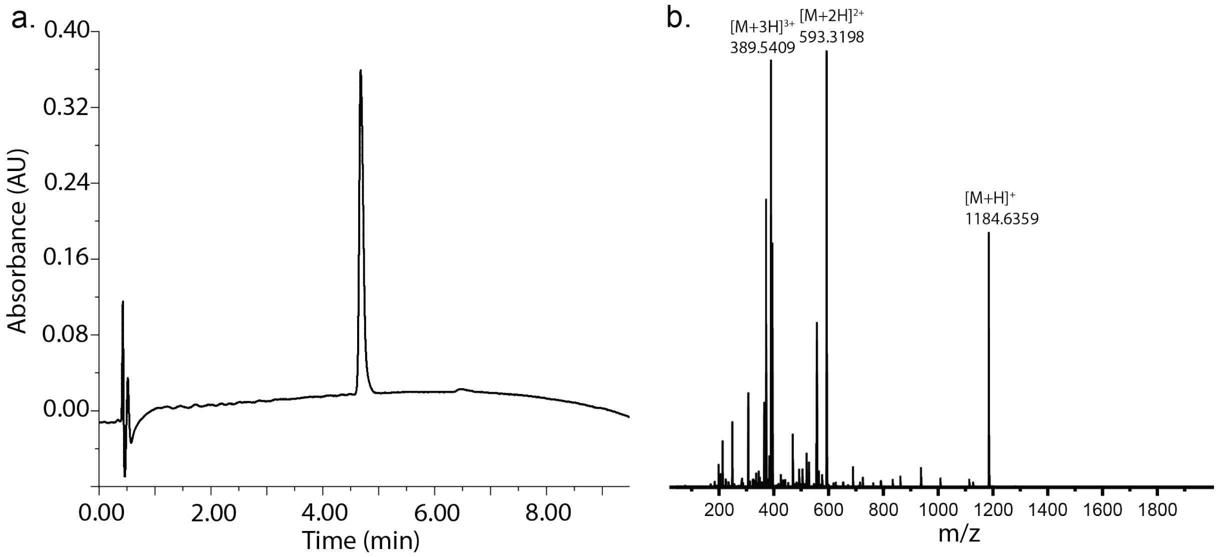

Figure **S5**. **a)** HPLC chromatogram (gradient from 5 to 85% ACN in water over 10 min at 2 ml/min, detection at 214 nm) and **b)** high-resolution mass spectrum for RhoB-Sar-Sar-ATCUN-Gly.

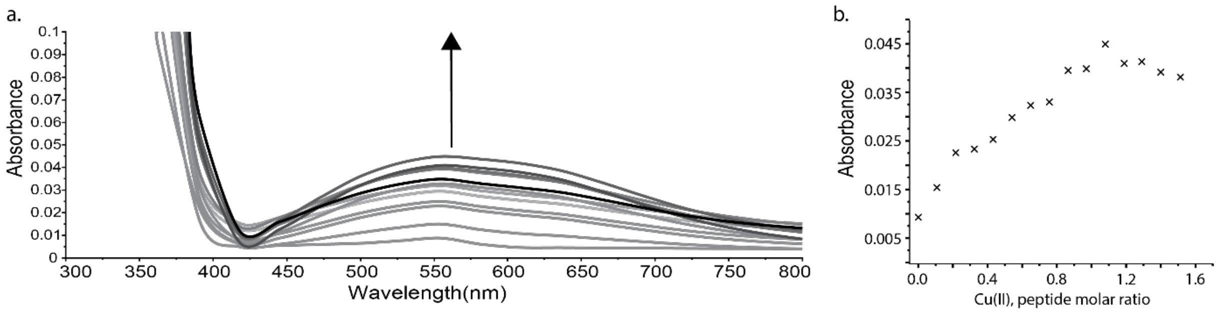

Figure **S5.** UV/Vis titration of Sar-ATCUN-Gly with $CuSO_4$ at pH 9.5. **a)** UV/Vis titration of Sar-ATCUN-Gly (1 mM, Tris buffer pH 9.5) at different concentrations of $CuSO_4$ (0 – 1.6 mM) and **b)** change in absorbance intensity as a function of the molar ratio of Cu(II) to Sar-ATCUN-Gly.

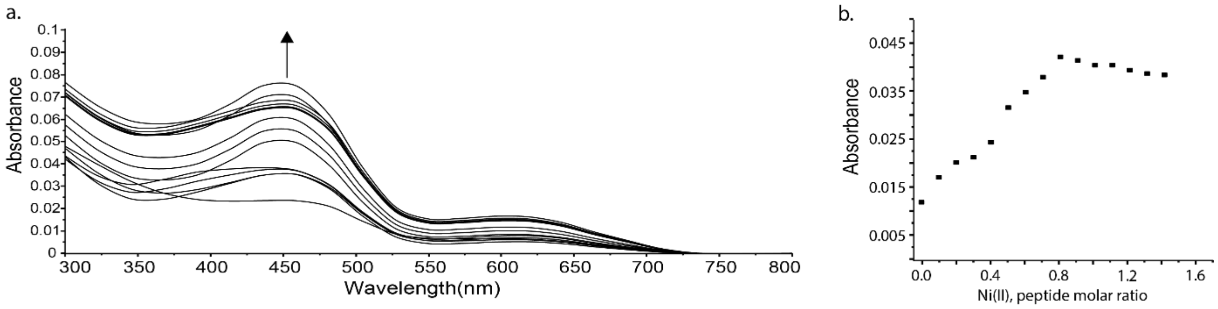

Figure **S6.** UV/Vis titration of Sar-ATCUN-Gly with $NiSO_4$ at pH 9.5. **a)** UV/Vis titration of Sar-ATCUN-Gly (1 mM, Tris buffer pH 9.5) at different concentrations of $NiSO_4$ (0 – 1.4 mM) and **b)** change in absorbance intensity as a function of the molar ratio of Ni(II) to Sar-ATCUN-Gly.

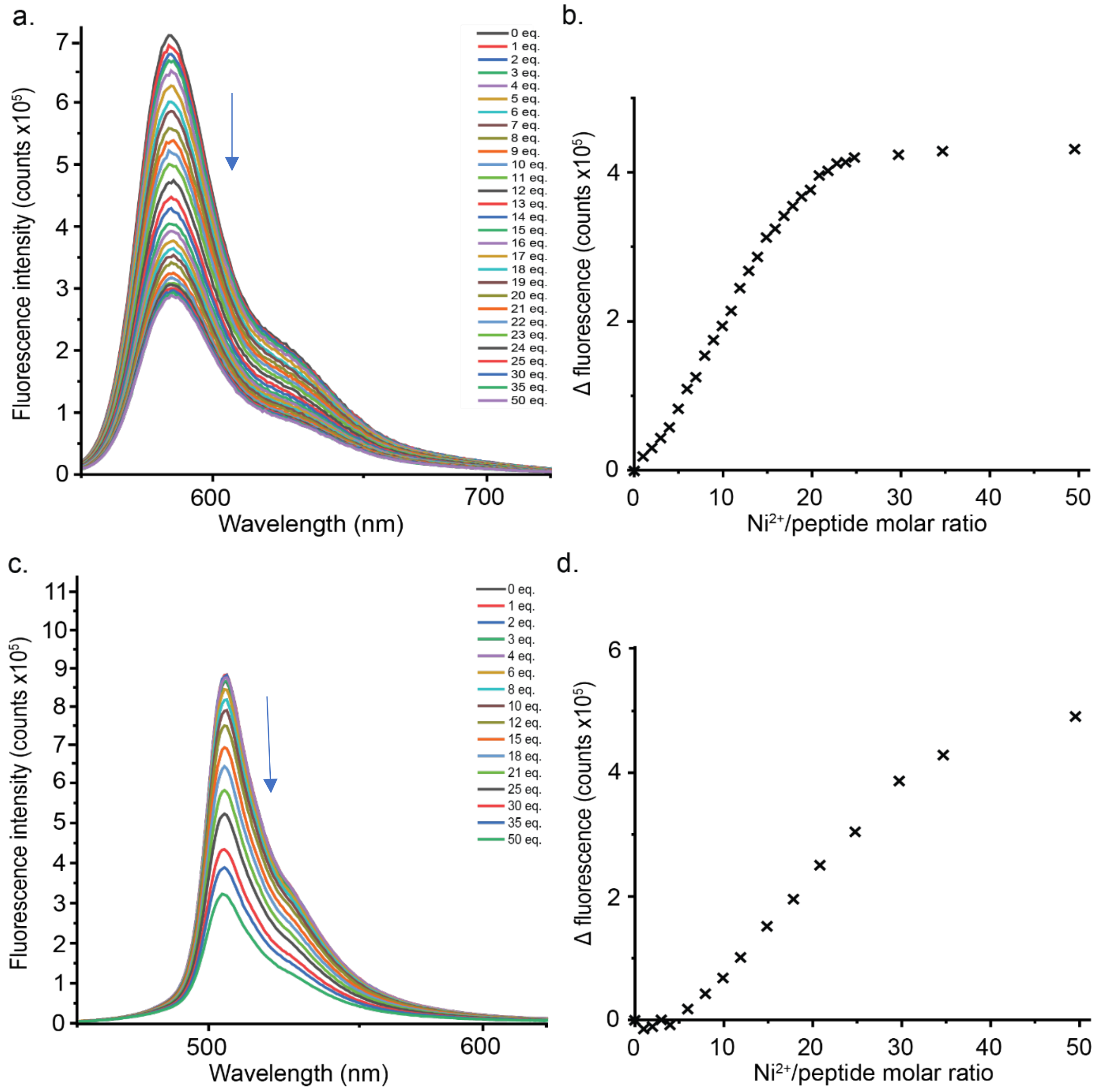

Figure **S7. a)** Decrease of fluorescence intensity of RhoB-Sar-ATCUN-Gly (5 µM, Tris buffer pH 9.5) upon addition of $NiCl_2$ (0-500 µM, in Tris buffer pH 9.5) and **b)** change of fluorescence intensitiy as a function of the molar ratio of Ni^{II} to RhoB-Sar-ATCUN-

Gly, **C.** decrease of fluorescence intensity of BODIPY-ATCUN-Gly (5 µM, Tris buffer pH 9.5) upon addition of $NiCl_2$ (0-500 µM, in Tris buffer pH 9.5), **d)** change of the fluorescence intensity as a function of the molar ratio of Ni^{II} to BODIPY-ATCUN-Gly.

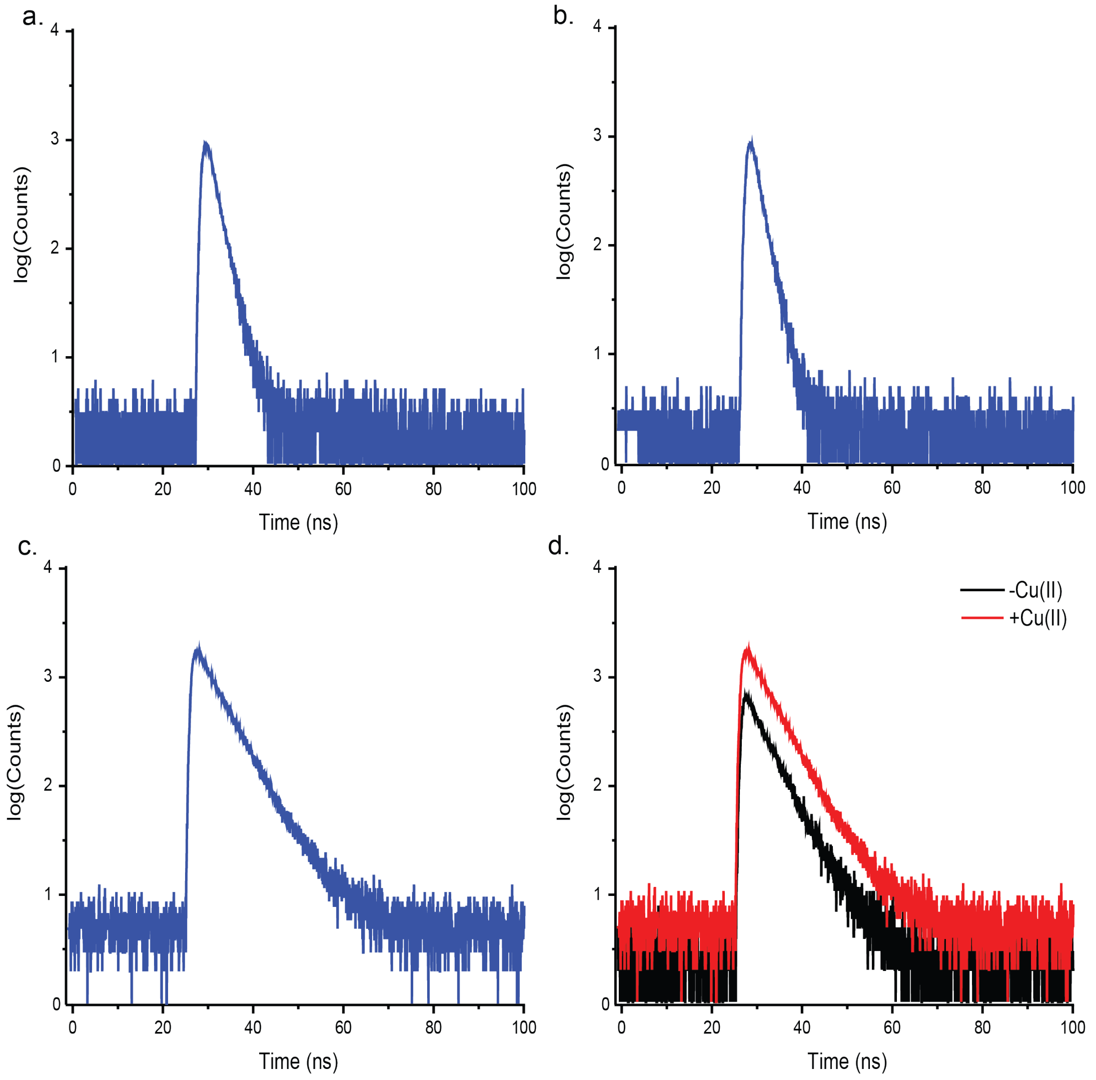

Figure **S8.** Fluorescence lifetime measurements of ATCUN peptides (5 µM in Tris buffer pH 9.5). **a)** RhoB-Sar-ATCUN-Gly, **b)** RhoB-Sar-Sar-ATCUN-Gly, **c)** BODIPY-ATCUN-Gly, **d)** BODIPY-ATCUN-Gly without Cu(II), black or with 250 µM Cu(II).

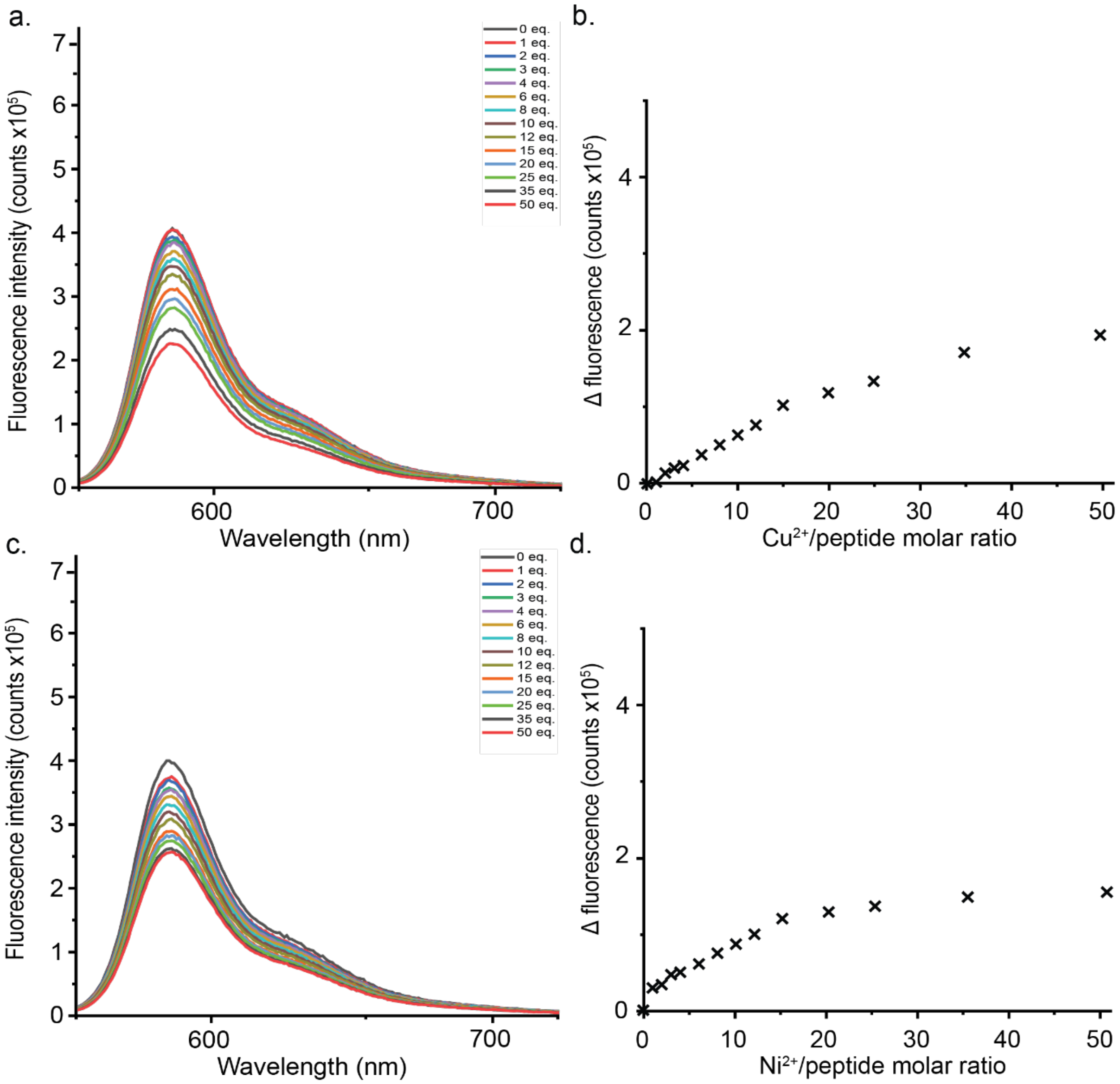

Figure **S9. a)** Decrease of fluorescence intensity of RhoB-Sar-Sar-ATCUN-Gly (5 µM, Tris buffer pH 9.5) upon addition of $CuSO_4$ (0-250 µM, in Tris buffer pH 9.5) and **b)** change of fluorescence intensitiy as a function of the molar ratio of Cu^{II} to RhoB-Sar-Sar-ATCUN-Gly, **c)** decrease of fluorescence intensity of RhoB-Sar-Sar-ATCUN-Gly (5 µM, Tris buffer pH 9.5) upon addition of $NiCl_2$ (0-500 µM, in Tris buffer pH 9.5), **d)** change of the fluorescence intensity as a function of the molar ratio of Ni^{II} to RhoB-Sar-Sar-ATCUN-Gly.

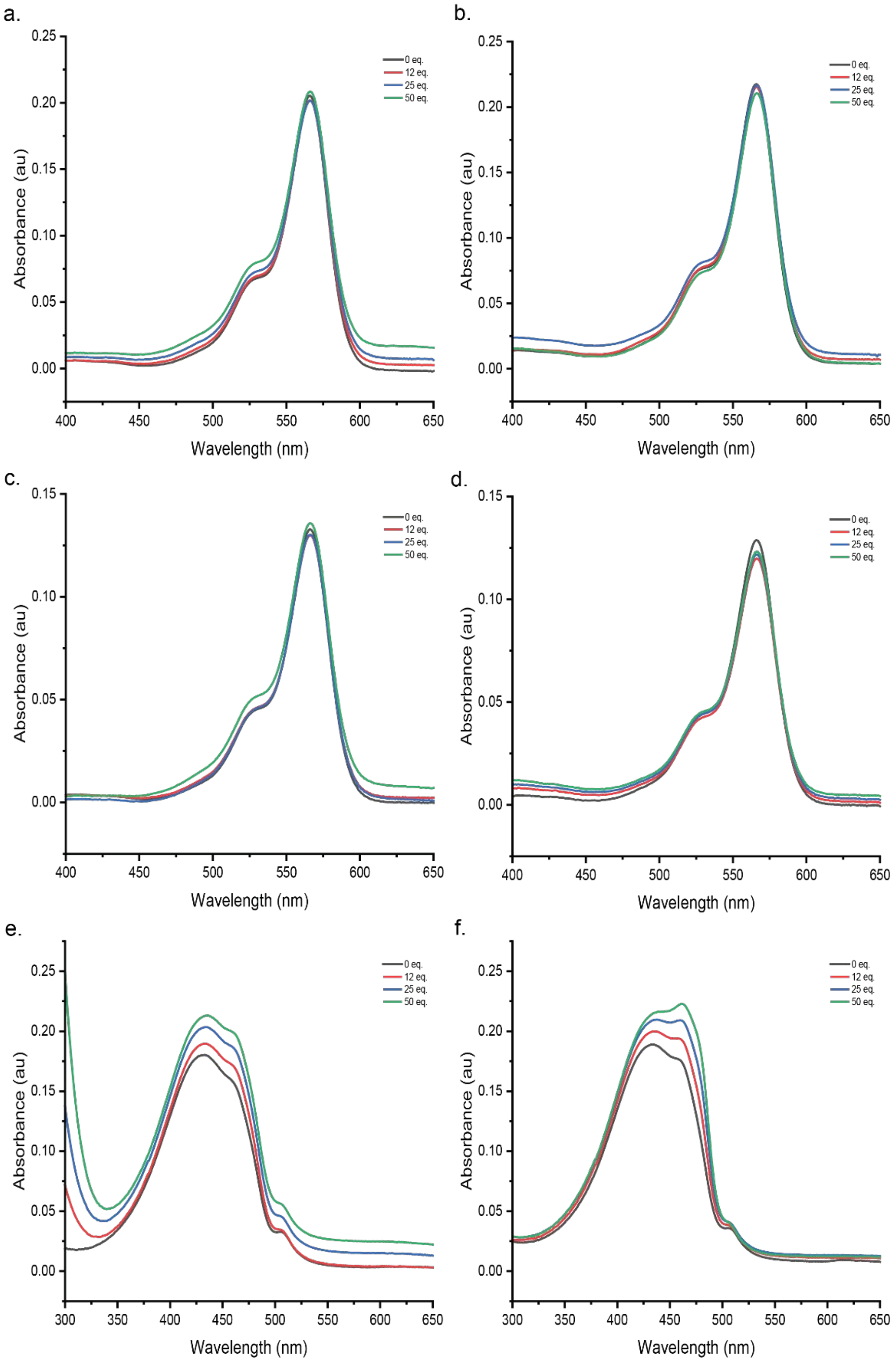

Figure **S10.** UV/Vis spectra upon addition of Cu(II) and Ni(II) (0-250) to ATCUN peptides (5 µM) during fluorescence measurements. **a)** RhoB-Sar-ATCUN-Gly with Cu^{II}, **b)** RhoB-Sar-ATCUN-Gly with Ni^{II}, **c)** RhoB-Sar-Sar-ATCUN-Gly with Cu^{II}, **d)** RhoB-Sar-Sar-ATCUN-Gly with Ni^{II}, **e)** BODIPY-ATCUN-Gly with Cu^{II} and **f)** BODIPY-ATCUN-Gly with Ni^{II}.

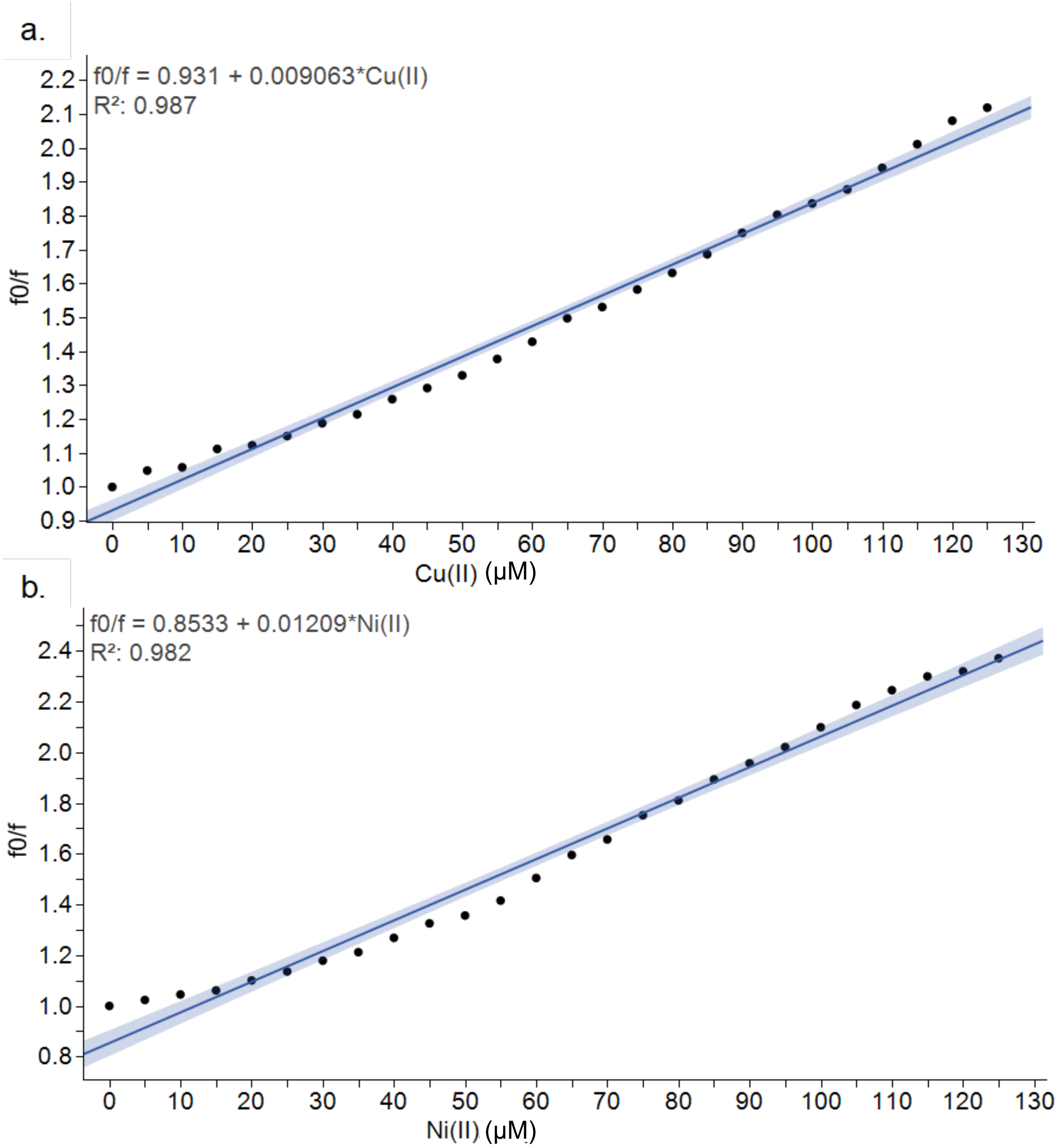

Figure **S11.** Stern-Volmer plots that present the binding curve between RhoB-Sar-ATCUN-Gly (5 µM in Tris buffer pH 9.5) and **a)** Cu(II) and **b)** Ni(II) over a concentration range of 0 to 125 µM.

a.

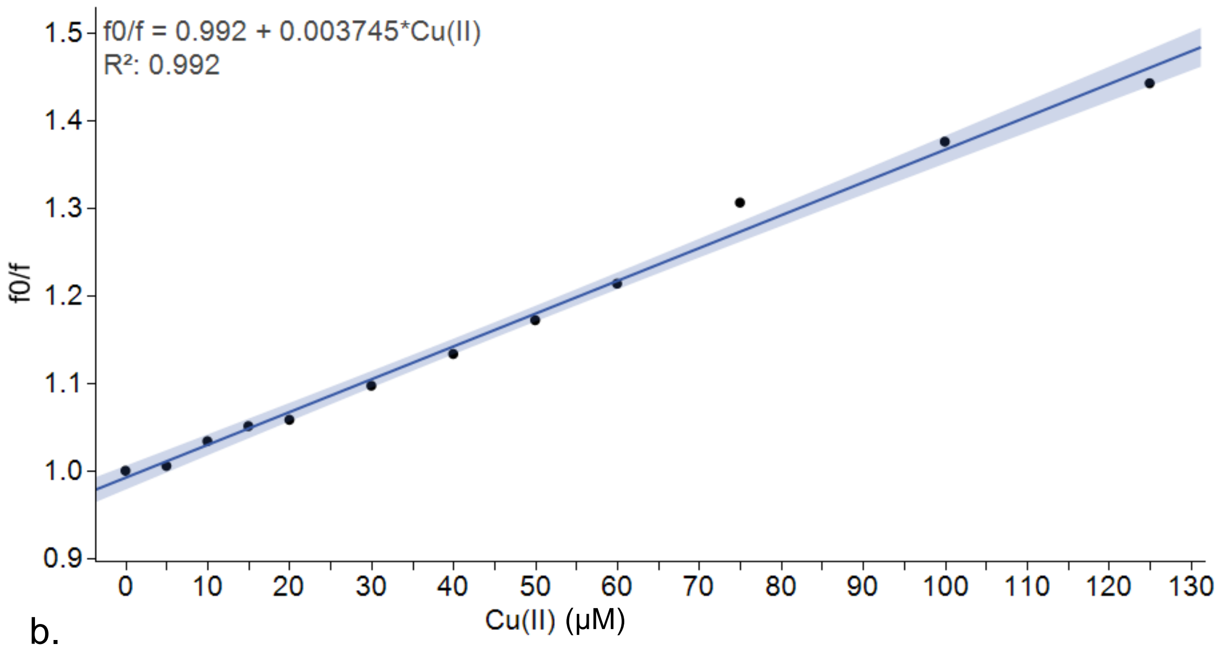

b.

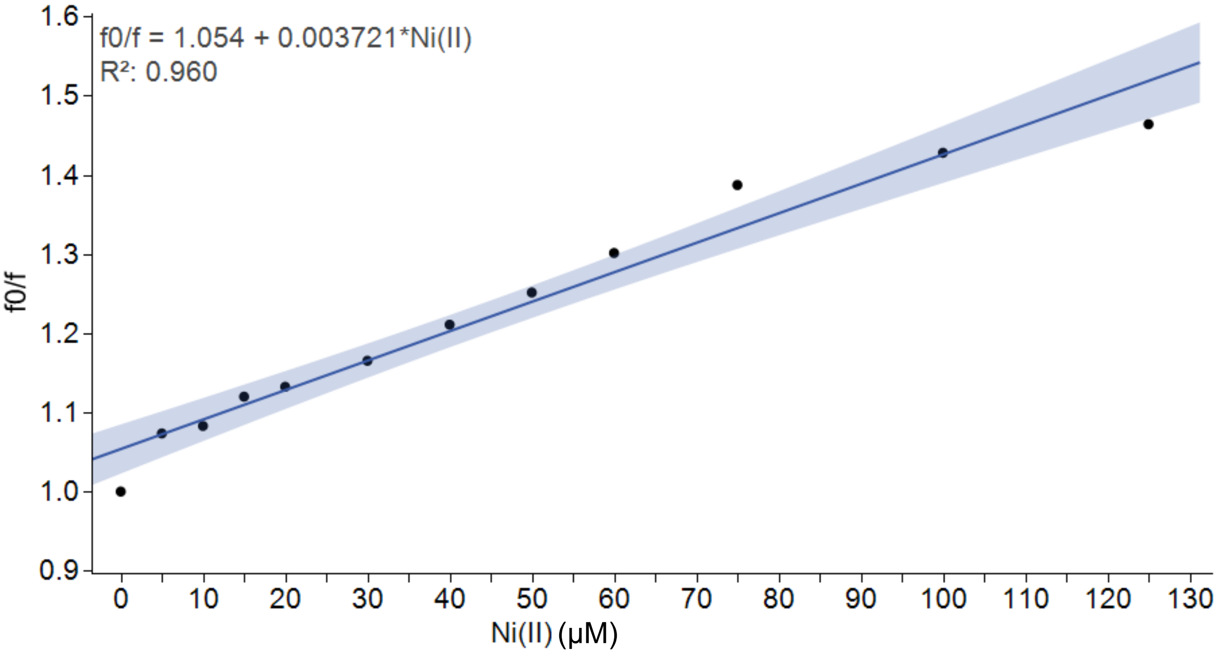

Figure **S12.** Stern-Volmer plots that present the binding curve between RhoB-Sar-Sar-ATCUN-Gly (5 μM in Tris buffer pH 9.5) and **a)** Cu(II) and **b)** Ni(II) over a concentration range of 0 to 125 μM.

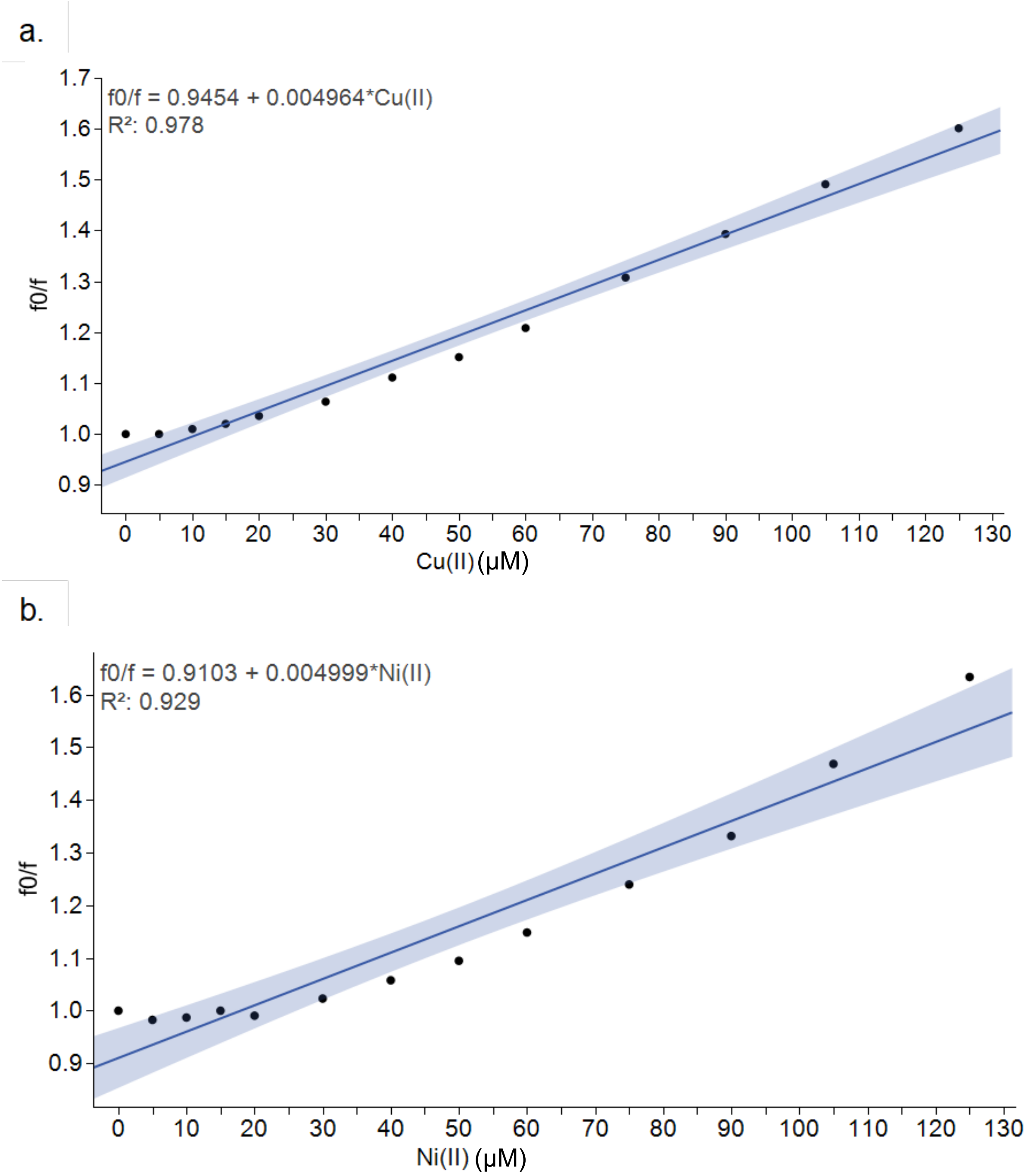

Figure **S13.** Stern-Volmer plots that present the binding curve between BODIPY-ATCUN-Gly (5 μM in Tris buffer pH 9.5) and **a)** Cu(II) and **b)** Ni(II) over a concentration range of 0 to 125 μM.

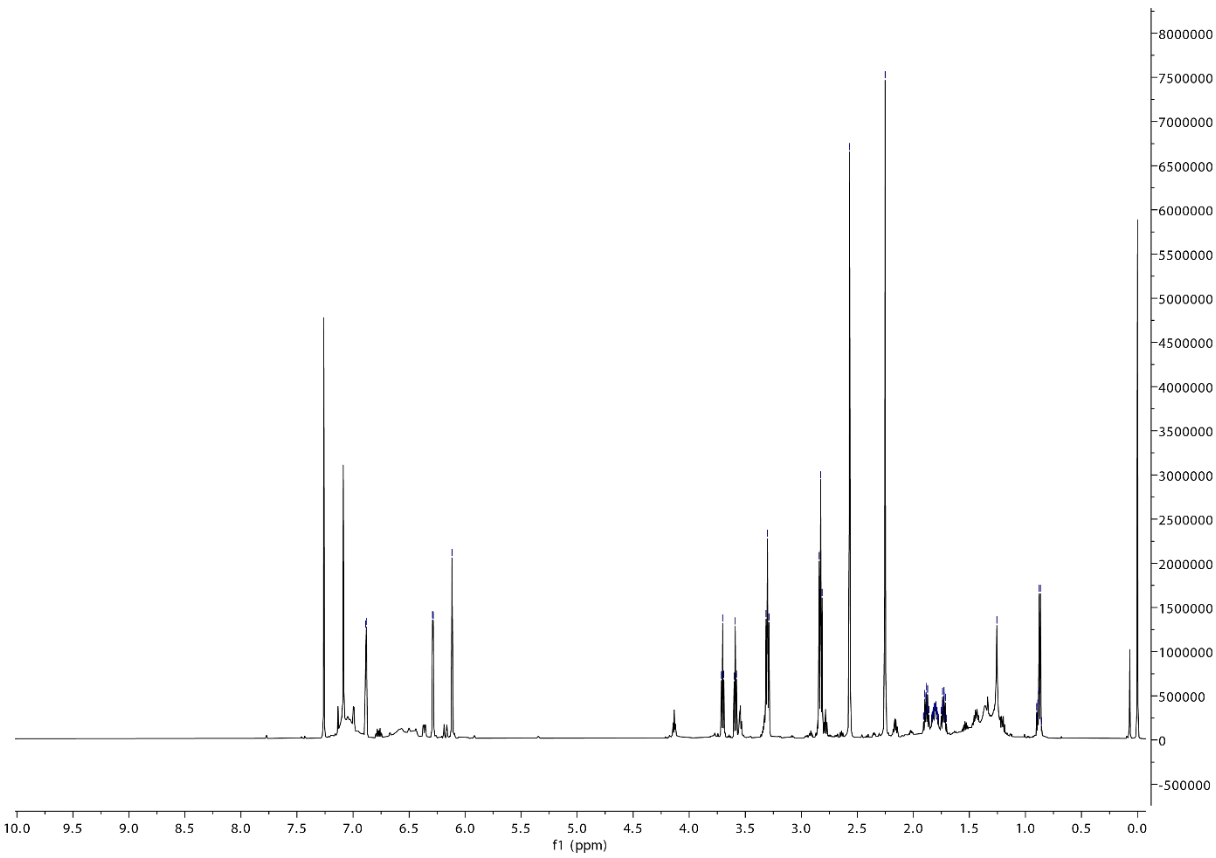

Figure **S14.** ^{1}H-NMR spectrum of BODIPY-FL-propanoic acid.
